# Parenting Styles and Parent–Adolescent Relationships: The Mediating Roles of Behavioral Autonomy and Parental Authority

**DOI:** 10.3389/fpsyg.2018.02187

**Published:** 2018-11-13

**Authors:** Xinwen Bi, Yiqun Yang, Hailei Li, Meiping Wang, Wenxin Zhang, Kirby Deater-Deckard

**Affiliations:** ^1^Department of Psychology, Shandong Normal University, Jinan, China; ^2^Department of Business, Shandong Normal University, Jinan, China; ^3^Department of Psychological and Brain Sciences, University of Massachusetts, Amherst, MA, United States

**Keywords:** parenting style, parent–adolescent relationship, behavioral autonomy, parental authority, gender

## Abstract

The parent–adolescent relationship has been a classic research topic, and researchers have found that parenting styles (e.g., authoritative, authoritarian) are closely related to various qualities of parent-adolescent relationships (e.g., cohesion, conflict). However, little empirical work has addressed how these variables correlate with each other in mainland China, nor has prior research addressed internal psychological mechanisms. The present study investigated the associations between parenting styles and parent–adolescent relationship factors, examined the mediating effects of adolescents’ expectations of behavioral autonomy and beliefs about parental authority, and explored whether adolescent gender moderated these effects. Results from a sample of 633 Chinese adolescents (7th grade: *M*_age_ = 13.50 ± 0.62 years, 9th grade: *M*_age_ = 15.45 ± 0.67 years, 11th grade: *M*_age_ = 17.30 ± 0.75 years) suggested similar levels of parent–adolescent conflict frequency for all parenting styles. However, for parent–adolescent conflict intensity, youth of neglectful and authoritarian parents reported higher levels compared to those with indulgent parents. The highest levels of cohesion with both parents were reported by adolescents with authoritative parents, followed by indulgent, authoritarian and neglect parenting styles. Cohesion with mothers for youth with authoritative or indulgent mothers was higher for girls than boys. Adolescents’ expectation for behavioral autonomy mediated the links between parenting style and conflict, whereas adolescents’ beliefs about the legitimacy of parental authority mediated the links between parenting style and cohesion; some of these mediating effects differed by gender. Findings highlight the importance of studying potential effects of adolescents’ values and attitudes within the family system in specific cultural contexts.

## Introduction

Variations in parenting styles and parent–child relationship qualities are long-standing research topics in developmental and family psychology. Previous research has shown that parenting styles are critical family context factors which are closely related to parent–adolescent relationships (Shek, 2002). Despite the large number of studies on the associations between parenting styles and parent–adolescent relationships, existing research mainly has focused on the direct effects of parenting styles on parent-adolescent relationships, while the underlying mechanisms through which parenting styles are associated with parent–adolescent relationships have seldom been examined. The present study examined the possible mediating effects of adolescents’ expectations for behavioral autonomy and beliefs in the legitimacy of parental authority, on the link between parenting style differences and variability in relationship conflict and cohesion, in a sample of youth from mainland China. We also tested whether the direct and mediated effects differed for girls and boys.

### Parenting Styles and Parent–Adolescent Relationships

Parenting style is defined as a constellation of parents’ attitudes and behaviors toward children and an emotional climate in which the parents’ behaviors are expressed (Darling and Steinberg, 1993). In the field of parenting, Maccoby and Martin’s (1983) and Baumrind’s (1991) typological approach of conceptualizing parenting has had a tremendous impact. They classified parenting into four types based on responsiveness and demandingness (Maccoby and Martin, 1983; Baumrind, 1991). Authoritative parenting style is characterized as high in responsiveness and demandingness. Authoritative parents provide not only support and warmth, but also clearly defined rules and consistent discipline (Baumrind, 1991). Authoritarian parenting style is characterized as low in responsiveness but high in demandingness. Parents of this style tend to use hostile control or harsh punishment in an arbitrary way to gain compliance, but they seldom provide explanation or allow verbal give-and-take. Indulgent parenting style is characterized as low in demandingness but high in responsiveness. Indulgent parents are responsive to their children and satisfy children’s needs, but they fail to set proper disciplinary, exhibit behavioral control, or make demands for mature behaviors. Finally, neglectful parenting style is characterized as low in responsiveness and demandingness. Neglectful parents are parent-centered and they are seldom engaged in child rearing practices. They neither provide warmth nor set rules for their children.

Adolescence is a critical developmental period that requires parents and youth to renegotiate their relationships (Laursen and Collins, 2009). Existing research has shown that variation in parenting styles is related to differences in parent-adolescent relationship features. Overall, most studies with Western samples have consistently found that authoritative parenting style is associated with higher levels of parent–adolescent cohesion (Nelson et al., 2011) and lower levels of conflict frequency (Smetana, 1995), conflict intensity (Smetana, 1995), and total conflict (McKinney and Renk, 2011). In contrast, an authoritarian parenting style is associated with lower cohesion (McKinney and Renk, 2011) and higher conflict frequency (Smetana, 1995; Sorkhabi and Middaugh, 2014), intensity (Smetana, 1995), and total conflict (McKinney and Renk, 2011). For instance, in a sample of American adolescents, Smetana (1995) found that more frequent and intense conflicts were predicted by more authoritarian parenting and less authoritative parenting. Similarly, Sorkhabi and Middaugh (2014) analyzed data from American adolescents who had Asian, Latino, Arab, European or other ethnic background. They found that adolescents of authoritative parents reported less conflict than those with authoritarian parents.

Most previous research on the associations between parenting styles and parent-adolescent conflict and cohesion focused on one or the other (e.g., Smetana, 1995; Nelson et al., 2011; Sorkhabi and Middaugh, 2014). However, conflict is not the opposite of cohesion, nor are increases over time in one necessarily associated with decreases in the other (Zhang et al., 2006). To comprehensively understand the links between parenting styles and these two aspects of the parent-adolescent relationship, both should be examined. Also, most previous research seldom distinguished conflict frequency and intensity or examined them simultaneously. Conflict frequency refers to how often conflict occurs, whereas conflict intensity refers to the magnitude of emotional arousal that occurs during conflict. Prior research on these two aspects of conflict has yielded mixed results. For example, Smetana (1995) found that parenting styles’ links with conflict frequency and intensity were very similar. In contrast, Assadi et al. (2011) reported that frequency was lower for authoritative parents and higher for authoritarian parents—but only authoritative parenting was linked to intensity. Thus, conflict intensity and frequency both should be examined.

Another major gap in the literature is that few of the relevant prior studies examined all four parenting styles. We know of only one American study (of adolescent substance abusers) that examined conflict, cohesion, and all four parenting styles (Smith and Hall, 2008). Actually, it’s also important to explore the relationships between indulgent and neglectful parenting style and parent–adolescent conflict and cohesion. Especially, neglectful parenting style which is characterized as disengaged from child rearing process may be destructive to parent–adolescent relationships. Thus, in light of the gaps in literature identified above, our first major aim was to explore the associations between all four parenting styles and parent–adolescent conflict (frequency and intensity) and cohesion. Based on prior evidence, we hypothesized that conflict (frequency and intensity) would be highest, and cohesion lowest, for youth with authoritarian parents—and conflict lowest and cohesion highest for adolescents with authoritative parents.

### Adolescent Autonomy and Beliefs About Parental Authority

In spite of the numerous prior studies of the link between parenting style and parent–adolescent relationship features, there are surprisingly few that have tested mechanisms that might account for the link. We also addressed this gap in the current study. According to Darling and Steinberg’s (1993) integrative model, parenting styles affect adolescents’ outcomes by changing the degree to which adolescents accept their parents’ attempts to socialize them. When parents use specific styles to rear children, adolescents are not just passive social beings, but play an active role in shaping the parent–adolescent relationship and in interpreting parenting behavior, in ways that influence their own outcomes. Particularly important to this psychological process are adolescents’ attitudes about behavioral autonomy and the legitimacy of parental authority (Darling et al., 2007).

### Adolescents’ Expectation for Behavioral Autonomy

Autonomy, in contrast to forced behavior, reflects actions that arise from the agency of the self rather than others (Chen et al., 2013). Variations in parenting style are associated with individual differences in adolescents’ autonomy beliefs. Authoritative parenting has been shown to be the most beneficial to youth, with regard to fostering healthy normative development of autonomy (Baumrind, 1991). In contrast, authoritarian parents provided too much strictness and supervision for their children, while indulgent and neglectful parents provided insufficient monitoring and guidance. Adolescents with non-authoritative parents are more likely to desire for more behavioral autonomy which is not satisfied in an appropriate way (Bush and Peterson, 2013). It is important to note, however, that not all studies find authoritative parenting to be optimal for youth autonomy—differences in findings that may be due to the sample characteristics or measures being used (e.g., Darling et al., 2005; Chan and Chan, 2009).

The development of adolescents’ autonomy, in turn, can have effects on parent–adolescent relationship features. Parents and adolescents expect increasing autonomy with age, but adolescents typically demand autonomy earlier than their parents are ready to grant it (Jensen and Dost-Gözkan, 2015; Pérez et al., 2016). Adolescents’ desire for more autonomy than their parents wish to grant them prompts youth to exert more control of their own affairs, and to be more critical of their parents’ control behaviors—a pattern that causes conflict and reduces cohesion (Fuligni, 1998; Zhang and Fuligni, 2006).

### Adolescents’ Beliefs About Parental Authority

In addition to developmental changes in autonomy, adolescence also is a period of youths’ changes in attitudes about parental authority—specifically, the extent to which parental assertion of control is seen as an appropriate extension of their role (Darling et al., 2008). Compared to other parenting styles, authoritative parents have children and adolescents who are more likely to endorse the legitimacy of parental authority (Smetana, 1995; Darling et al., 2005; Trinkner et al., 2012). In contrast, authoritarian parents tend to define issues as falling into parental jurisdiction too rigidly, and indulgent and neglectful parents define these too permissively (Smetana, 1995; Baumrind, 2005). In those cases, adolescents and parents may be deprived of opportunities to debate and negotiate appropriate boundaries, which in turn can lead youth to question and doubt the legitimacy of parental authority.

Attitudes about legitimacy of authority are also linked with parent–adolescent relationship features. Adolescents’ endorsement of parental authority is associated with greater cohesion and less conflict with parents (Zhang et al., 2006; Jensen and Dost-Gözkan, 2015)—in one study, a pattern found in Mexican, Chinese, Filipino, and European background families (Fuligni, 1998).

In sum, there are well-established links between parenting style, adolescents’ beliefs (specifically, about autonomy and parental authority), and parent-adolescent relationship qualities. However, these different constructs have not been examined all together in one study. In addition, although previous studies have examined the associations between parenting styles and parent-adolescent relationships, there was no research that examined whether adolescents’ expectation for behavioral autonomy and endorsement of parental authority mediated these associations. Thus, our second aim was to test the hypothesis that expectations for behavioral autonomy and beliefs in the legitimacy of parental authority both would mediate the link between parenting styles and parent-adolescent conflict and cohesion.

### The Role of Adolescent Gender

The third and final aim of the current study was to examine potential gender differences in the relationships between parenting styles, parent-adolescent conflict and cohesion, adolescents’ expectation for behavioral autonomy and endorsement of parental authority. There is reason to expect differences to be found, although results may differ depending on the parenting styles and parent-adolescent relationship features in question. For instance, Shek (2002) reported an association between parental negativity and greater parent-adolescent conflict, only for girls. These differences may reflect distinct socialization goals for boys and girls, with girls oriented more toward family relationships and compliance, and boys oriented toward autonomy and self-reliance (Shek, 2002; Zhang et al., 2006). Based on previous research, we expected to find stronger associations between parenting style and parent–adolescent relationship features for girls compared to boys. However, given the lack of prior research on beliefs about autonomy and parental authority as mediators, we had no hypotheses regarding gender as moderator of those mediating effects.

### Chinese Cultural Context

As a final point, another rationale for the current study was to address the dearth of research on mainland Chinese families published in the international literature. The existing evidence is almost completely dominated by studies of families from Western industrial nations, even though mainland China has the single largest population of children and adolescents in the world—in 2016, 13% or nearly one in eight of the globe’s 0–14-year-olds (World Bank, 2017). We know of only one relevant published study of parenting styles and parent–adolescent relationships, which found that authoritative mothers exhibited the highest levels, and authoritarian mothers the lowest levels, of mother-adolescent cohesion (Zhang et al., 2017). Adding to the literature base to include evidence from non-Western nations such as China, serves to extend and deepen knowledge of parent-adolescent relationship processes.

Studying mainland Chinese families also offers a unique opportunity for examining family processes because its culture is so distinct from Western contexts. Two features in particular stand out. First, China has been unique in the world in its “one child policy” implemented by the government from 1979 until 2016. This led to a significant change in the family, often described as the “4-2-1” family structure (four grandparents, two parents, and one child). In this context, the relationships between parenting styles and parent–adolescent conflict and cohesion in China may be different from those in Western cultures. Second, Chinese culture is rooted in Confucianism, which emphasizes collectivist values such as conforming to social norms, submission to authority, establishing strong relationships with others, and avoiding confrontation (Peterson et al., 2005). In this strict hierarchical framework, individuals’ requests for autonomy and any behaviors that potentially threaten group harmony are discouraged, whereas great respect for parental authority is highly valued (Fuligni, 1998). Furthermore, some research has shown that autonomy and authority beliefs among adolescents covary with family relationship features in different ways depending on cultural context. For example, one study reported that conflict intensity with mothers was greater for adolescents with lower respect for parental authority in African American and Latina, but not European American, families (Dixon et al., 2008). Thus, there is a need to broaden the diversity of samples in this literature, to better understand which aspects of the relevant family processes operate similarity, or differently, in distinct cultural contexts.

In sum, the current study addressed three aims in a mainland China sample of families: (1) to explore the links between four parenting styles and parent-adolescent relationship conflict (frequency and intensity) and cohesion, including testing the hypothesis that conflict would be highest and cohesion lowest for authoritarian parents, conflict lowest and cohesion highest for authoritative parents; (2) to test the hypothesis that the links between parenting style and parent–adolescent relationship features would be statistically mediated by adolescents’ autonomy expectations and beliefs regarding parental authority; and (3) to test the hypothesis that the links between parenting style and relationship features (explored in Aim 1) would be stronger for girls than for boys—and to also explore gender differences in the mediating effects (hypothesized in Aim 2).

## Materials and Methods

### Participants and Procedure

A total of 633 students (48.5% females, in line with the proportion found in the Chinese population) in the 7th (*M*_age_ = 13.50 ± 0.62 years), 9th (*M*_age_ = 15.45 ± 0.67 years) and 11th (*M*_age_ = 17.30 ± 0.75 years) grades of four schools in Jinan, the capital of Shandong Province in Middle Eastern China, completed self-report questionnaires. Because of the implementation of one child policy in mainland China, 90 percent of them were only children.

Surveys were completed in class through group administration; students were asked not to communicate with each other while completing the survey. Research staff members administered the surveys to the class by introducing the purpose of this study and the voluntary nature of participation, reading instructions and answering any questions that arose during the data collection period. All participants gave written informed consent. Additionally, all parents of participants were notified about the research and were given the opportunity to withdraw their children from study participation. All parents gave written informed consent to allow their children to participate in this study. The Institutional Review Board of Shandong Normal University approved this study procedures.

### Measures

#### Parenting Styles

Parenting styles were assessed using the Chinese version of Steinberg et al.’s (1994) parenting styles questionnaire (Long et al., 2012). Two subscales comprise the measure of parenting: acceptance/involvement and strictness/supervision. The acceptance/involvement subscale (α = 0.84) was the average of 15 items that were used to assess responsive, loving and involved parenting (e.g., “I can count on my parents to help me out if I have some kind of problem.”). The strictness/supervision subscale (α = 0.78) was the average of 12 items that was used to assess monitoring and supervision (e.g., “How much do your parents try to know where you go out at night”). The adolescents were required to indicate the strength of endorsement using a 5-point scale ranging from 1 (*completely disagree*) to 5 (*completely agree*) for each item. Confirmatory factor analysis indicated that the measurement of parenting styles (as well as endorsement of parental authority, expectations for behavioral autonomy and parent-adolescent conflict and cohesion) had acceptable construct validity and strong measurement invariance across gender (see Online Supplementary Materials and Supplementary Table S1).

#### Endorsement of Parental Authority

Adolescents’ beliefs about the legitimacy of parental authority were assessed using Chinese version of Smetana’s (1988) questionnaire (Zhang and Fuligni, 2006). Students were presented with a list of 13 topics as individual items such as curfew, choosing clothes, and choosing friends, and were asked whether father or mother could make a rule about each topic. Responses for each topic/item were coded on a 4-point scale ranging from 1 (*It’s not OK*) to 4 (*It’s completely OK*). These were averaged separately for mother (α = 0.84) and father (α = 0.86).

#### Expectations for Behavioral Autonomy

Adolescents’ expectation for behavioral autonomy was measured based on the questionnaire from Fuligni (1998). Students were presented with a list of 12 behaviors (e.g., “watch as much TV as you want”). Adolescents then indicated the degree of expectation for each item using a 4-point scale ranging from 1 (*expect heavily*) and 4 (*not expect at all*) (α = 0.86). In order to achieve consistency across all instruments so that a high score would reflect a high level of the variable being measured, these entries were reversed score so that 1 was recoded as 4, 2 as 3, 3 as 2, and 4 as 1.

#### Parent–Adolescent Conflict

Adolescents’ perceptions of the incidence and intensity of conflict with their mothers and fathers were measured by the Chinese version of Issues Checklist (Prinz et al., 1979; Zhang and Fuligni, 2006). Students indicated whether the 16 specific topics (e.g., chores, cursing) were discussed or not with their parents within the past 2 weeks (using a binary scale, *yes* or *no*). Then, for each endorsed topic of discussion, adolescents reported the conflict intensity of the discussion of each topic, using a 5-point scale that varied from 1 (*very calm*) to 5 (*very angry*). To be consistent with previous research (e.g., Fuligni, 1998), conflict frequency was computed by summing the number of discussions rated as containing anger (2 or greater on the 5-point scale). Conflict intensity was obtained by averaging adolescents’ rating on those items that were discussed (mother: α = 0.72, father: α = 0.73).

#### Parent–Adolescent Cohesion

Adolescents completed the cohesion subscale of the Chinese version of Family Adaptation and Cohesion Evaluation Scales (FACES) II inventory separately for each parent (Olson et al., 1979; Zhang and Fuligni, 2006). This scale included 10 items (e.g., “My mother [father] and I feel very close to each other”). Students’ perception of cohesion with parents was rated on a 5-point scale ranging from 1 (*almost never*) to 5 (*almost always*), separately for mother (α = 0.82) and father (α = 0.79).

#### Controlled Variables

Grade and socioeconomic status (SES) were controlled for this study. The SES score was computed by averaging the standardized education and occupation of both parents. Parents’ education was coded as 1 = equal to or below primary school, 2 = junior high school, 3 = senior high school, 4 = some college or above. The occupation was coded as 1 = peasant or jobless, 2 = blue collar, 3 = professional or semiprofessional. In terms of parents’ educational level, approximately 0.8% of the mothers and 0.3% of fathers had completed primary school education or less, and 38.5% of mothers and 57.1% of fathers had a college or university degree. The remainder had either a junior high school education (7.6% of mothers and 5.5% of fathers) or a senior high school education (48.2% of mothers and 31.5% of fathers). The occupational status of mothers and fathers, respectively, was as follows: 6.2 and 2.7% were peasants or jobless, 28.4 and 23.4% had blue collar position, and 64.9 and 73.6% held a professional or semiprofessional occupation.

## Results

### Descriptive Statistics

We used Harman’s single factor test to check the common method bias. The results showed that 30 factors emerged with eigenvalues greater than 1.0 and the first factor accounted for only 16.53% of total variance. Since more than one factor emerged and the first factor did not account for the majority of the variance (Podsakoff and Organ, 1986), common method bias was not a serious concern in the present study.

Cluster analysis with K-means method was used to identify the four parenting styles. Instead of defining parentings styles *a priori* based on subjective cut-off scores (Steinberg et al., 1994), in cluster analysis families are grouped according to their scores on various parenting characteristics (Henry et al., 2005). To validate the cluster solution, we reanalyzed the data with a different cluster method — a hierarchical cluster analysis (Henry et al., 2005; Hoeve et al., 2007). We repeated the hierarchical cluster analysis ten times, applying the standardized Euclidian Distance method as a distance measure and using Ward’s algorithm. The cross validation procedure (Mandara, 2003) result in moderate agreements (*k* = 0.71, range: 0.67–0.75).

To label the four groups, we examined the parenting styles by computing a one-way ANOVA on the standardized scores of parenting dimensions with the clusters serving as the factors. The result revealed that the clustering variables significantly differed between the parenting dimensions [acceptance/involvement: *F*(3,608) = 472.58, *p* < 0.001, η^2^ = 0.70; strictness/supervision: *F*(3,608) = 280.35, *p* < 0.001, η^2^ = 0.58]. Authoritative parents were those who scored high on both dimensions (acceptance/involvement: *z* = 0.95, strictness/supervision: *z* = 0.76), whereas neglectful parents scored low on both dimensions (acceptance/involvement: *z* = -1.45, strictness/supervision: *z* = -1.06). Authoritarian parents scored low on acceptance/involvement (*z* = -0.61) but high on strictness/supervision dimension (*z* = 0.50), whereas indulgent parents scored high on acceptance/involvement (*z* = 0.15) but low on strictness/supervision dimension (*z* = -0.77).

Descriptive statistics for study variables are presented in Table 1, and bivariate correlations are presented in Table 2. Regarding descriptives, the following frequencies were found for the four parenting styles: 152 (24.0% of total sample) authoritarian; 200 (31.6%) authoritative; 83 (13.1%) neglectful; and 177 (28.0%) indulgent. The average scores of beliefs in parents’ authority and expectation for behavioral autonomy ranged from 2 to 3, which implied that adolescents reported medium level of endorsement of parental authority and autonomy expectations. The average scores of conflict frequency ranged from 2 to 4 and the average scores of conflict intensity ranged from 1 to 2, which suggested that adolescents reported low level of conflict frequency and intensity. Since the cohesion scored larger than 3 (except girls with neglectful parents), adolescents reported medium-high level of cohesion with parents.

**Table 1:** Means and standard deviations of all study variables except parenting styles.

	Indulgent		Neglectful		Authoritarian		Authoritative		Group differences
	Male	Female	Male	Female	Male	Female	Male	Female	
Beliefs about mother’s authority	2.37	2.16	2.09	1.99	2.37	2.48	2.75	2.71	Aut > Aun & Ind > Neg;
	(0.56)	(0.44)	(0.48)	(0.44)	(0.59)	(0.58)	(0.54)	(0.52)	Ind: M > F
Beliefs about father’s authority	2.39	2.12	2.00	1.94	2.34	2.43	2.73	2.70	Aut > Aun & Ind > Neg;
	(0.60)	(0.46)	(0.56)	(0.53)	(0.62)	(0.61)	(0.59)	(0.55)	Ind: M > F
Expectation for behavioral autonomy	2.52	2.45	2.71	2.67	2.50	2.34	2.24	2.14	Neg & Ind & Aun > Aut
	(0.68)	(0.57)	(0.67)	(0.59)	(0.66)	(0.66)	(0.69)	(0.52)	
Frequency of conflict with mother	3.18	3.52	3.93	4.33	3.63	4.33	3.63	3.05	No significant difference
	(3.18)	(2.86)	(2.86)	(3.65)	(3.06)	(3.02)	(3.25)	(2.88)	
Frequency of conflict with father	2.20	2.50	2.63	2.44	2.63	3.00	2.87	2.37	No significant difference
	(2.70)	(2.57)	(2.35)	(2.49)	(3.02)	(2.72)	(2.98)	(2.69)	
Intensity of conflict with mother	1.45	1.47	1.74	1.75	1.62	1.64	1.55	1.43	Neg & Aun > Ind;
	(0.43)	(0.42)	(0.61)	(0.65)	(0.55)	(0.52)	(0.53)	(0.40)	Neg > Aut
Intensity of conflict with father	1.42	1.47	1.79	1.59	1.65	1.61	1.54	1.42	Neg & Aun > Ind
	(0.42)	(0.49)	(0.88)	(0.66)	(0.82)	(0.67)	(0.61)	(0.44)	
Cohesion with mother	3.48	3.70	3.10	2.94	3.34	3.48	3.64	4.03	Aut > Ind > Aun > Neg;
	(0.50)	(0.51)	(0.65)	(0.70)	(0.54)	(0.67)	(0.56)	(0.55)	Ind & Aut: F > M
Cohesion with father	3.53	3.56	3.05	2.95	3.25	3.33	3.76	3.78	Aut > Ind > Aun > Neg
	(0.57)	(0.68)	(0.72)	(0.81)	(0.72)	(0.72)	(0.63)	(0.64)	


*Aut, authoritative parenting style; Aun, authoritarian parenting style; Neg, neglect parenting style; Ind, indulgent parenting style. M, male adolescents; F, female adolescents. Bonferroni *post hoc* tests were used.*

**Table 2:** Correlations for all study variables except parenting styles.

	1	2	3	4	5	6	7	8	9
(1) Beliefs about mother’s authority	–	0.91^∗∗∗^	–0.45^∗∗∗^	–0.19^∗∗∗^	–0.11	–0.19^∗∗^	–0.10	0.32^∗∗∗^	0.23^∗∗∗^
(2) Beliefs about father’s authority	0.89^∗∗∗^	–	–0.44^∗∗∗^	–0.14^∗^	–0.08	–0.14^∗^	–0.11	0.28^∗∗∗^	0.26^∗∗∗^
(3) Expectation for behavior autonomy	–0.62^∗∗∗^	–0.56^∗∗∗^	–	0.30^∗∗∗^	0.23^∗∗∗^	0.31^∗∗∗^	0.22^∗∗∗^	–0.27^∗∗∗^	–0.24^∗∗∗^
(4) Frequency of conflict with mother	–0.21^∗∗∗^	–0.19^∗∗∗^	0.25^∗∗∗^	–	0.73^∗∗∗^	0.81^∗∗∗^	0.58^∗∗^	–0.24^∗∗∗^	–0.06
(5) Frequency of conflict with father	–0.14^∗^	–0.14^∗^	0.19^∗∗^	0.70^∗∗∗^	–	0.60^∗∗∗^	0.74^∗∗^	–0.09	–0.10
(6) Intensity of conflict with mother	–0.29^∗∗^	–0.31^∗∗∗^	0.31^∗∗∗^	0.75^∗∗∗^	0.46^∗∗∗^	–	0.72^∗∗∗^	–0.35^∗∗^	–0.11
(7) Intensity of conflict with father	–0.23^∗∗^	–0.30^∗∗∗^	0.24^∗∗^	0.37^∗∗∗^	0.54^∗∗∗^	0.52^∗∗∗^	–	–0.16^∗∗^	–0.20^∗∗^
(8) Cohesion with mother	0.42^∗∗∗^	0.37^∗∗∗^	–0.22^∗∗∗^	–0.19^∗∗^	–0.15^∗∗^	–0.26^∗∗∗^	–0.18^∗∗^	–	0.40^∗∗∗^
(9) Cohesion with father	0.30^∗∗∗^	0.41^∗∗∗^	–0.15^∗∗^	–0.14^∗^	–0.15^∗∗^	–0.26^∗∗∗^	–0.35^∗∗∗^	0.45^∗∗∗^	–


*^∗^p < 0.05; ^∗∗^*p* < 0.01; ^∗∗∗^*p* < 0.001. Numbers above the diagonal refer to girls, and numbers below the diagonal refer to boys.*

Turning to correlations, although with a few exceptions, overall the adolescents’ higher expectation for behavioral autonomy was associated with greater frequency and intensity of conflict, and less cohesion. Adolescents’ stronger endorsement of the legitimacy of parental authority was associated with greater cohesion, but less frequent and intense conflict.

### Links With Parenting Styles

A series of 4 (parenting styles) × 2 (child gender) analyses of covariance was conducted to explore the links between four parenting styles and parent–adolescent relationships. At the same time, we also explored if adolescents’ expectation for behavior autonomy and endorsement of parental authority differed as a function of adolescents’ gender and parenting styles. SES and grade served as covariables.

For adolescents’ expectation for behavior autonomy, the main effect of parenting styles was significant [*F*(3,597) = 8.74, *p* < 0.001]. Bonferroni *post hoc* *t*-tests indicated that adolescents of authoritative parents reported the lower level of expectation for behavioral autonomy (*M* = 2.18, *SD* = 0.60) than adolescents of neglectful [*M* = 2.70, *SD* = 0.64, *t*(278) = 4.66, *p* < 0.001], indulgent [*M* = 2.48, *SD* = 0.62, *t*(371) = 3.75, *p* < 0.01] and authoritarian parents [*M* = 2.43, *SD* = 0.66, *t*(344) = 2.79, *p* < 0.05].

For legitimacy of parental authority, the main effect of parenting styles was significant [mother: *F*(3,597) = 30.26, father: *F*(3,597) = 29.62, *p*s < 0.001]. Adolescents of authoritative parents reported the highest endorsement of parental authority (mother: *M* = 2.73, *SD* = 0.53; father: *M* = 2.71, *SD* = 0.56), whereas adolescents of neglectful parents reported the lowest endorsement of parental authority (mother: *M* = 2.06, *SD* = 0.47; father: *M* = 1.98, *SD* = 0.54). Adolescents raised by authoritarian (mother: *M* = 2.42, *SD* = 0.59; father: *M* = 2.38, *SD* = 0.62) and indulgent parents (mother: *M* = 2.26, *SD* = 0.51; father: *M* = 2.25, *SD* = 0.55) reported endorsements of parental authority that were between the other two groups (mother: *t* > 2.86, *p* < 0.05; father: *t* > 3.52, *p* < 0.01). The interaction between gender and parenting styles also was significant [mother: *F*(3,597) = 2.53, *p* = 0.056; father: *F*(3,597) = 3.03, *p* < 0.05]. *Post hoc* probing revealed no gender difference for youth with authoritative, authoritarian and neglectful parents. In contrast, for youth with indulgent parents, boys reported greater endorsement of parental authority (mother: *M* = 2.37, *SD* = 0.56; father: *M* = 2.39, *SD* = 0.60) than did girls [mother: *M* = 2.16, *SD* = 0.44, *t*(171) = 2.62, *p* < 0.01; father: *M* = 2.12, *SD* = 0.46, *t*(171) = 3.52, *p* < 0.01].

Turning to intensity of conflict with parents, the main effect of parenting styles was significant [mother: *F*(3,595) = 7.49, *p* < 0.001; father: *F*(3,583) = 3.90, *p* < 0.01]. Adolescents of neglectful [mother: *M* = 1.74, *SD* = 0.62, *t*(253) = 3.99, *p* < 0.001; father: *M* = 1.73, *SD* = 0.81, *t*(245) = 2.58, *p* = 0.06] and authoritarian parents [mother: *M* = 1.63, *SD* = 0.54, *t*(320) = 3.01, *p* < 0.05; father: *M* = 1.63, *SD* = 0.75, *t*(313) = 2.49, *p* = 0.08] reported more intense conflict than those of indulgent parents (mother: *M* = 1.46, *SD* = 0.43; father: *M* = 1.45, *SD* = 0.46). In addition, adolescents of neglectful parenting also reported more intense conflict with mothers than those of authoritative parenting [*M* = 1.49, *SD* = 0.47, *t*(276) = 3.61, *p* < 0.01]. As for the frequency of conflict with parents, none of the effects was significant.

For cohesion, gender was significantly related to mother–child cohesion [*F*(1,597) = 9.07, *p* < 0.01], with greater cohesion found for daughters than sons (girls: *M* = 3.70, *SD* = 0.66; boys: *M* = 3.42, *SD* = 0.59). For mothers and fathers alike, there was a main effect of parenting styles [mother: *F*(3,597) = 37.53, father: *F*(3,597) = 26.49, *p*s < 0.001]. Adolescents of authoritative parents reported the highest level of cohesion (mother: *M* = 3.85, *SD* = 0.58; father: *M* = 3.77, *SD* = 0.63), followed by indulgent [mother: *M* = 3.59, *SD* = 0.52, *t*(371) = 4.20, *p* < 0.001; father: *M* = 3.55, *SD* = 0.63, *t*(371) = 3.15, *p* < 0.05], authoritarian [mother: *M* = 3.41, *SD* = 0.60, *t*(320) = 2.62, *p* = 0.05; father: *M* = 3.29, *SD* = 0.72, *t*(320) = 3.33, *p* < 0.01] and neglectful parents [mother: *M* = 3.05, *SD* = 0.67, *t*(227) = 4.78, *p* < 0.001; father: *M* = 3.02, *SD* = 0.75, *t*(227) = 2.94, *p* < 0.05]. Finally, the parenting style main effect for mothers was moderated by child gender [*F*(3,597) = 1.34, *p* < 0.01]. Cohesion was higher for girls than boys, only in authoritative [girls: *M* = 4.03, *SD* = 0.55; boys: *M* = 3.64, *SD* = 0.56, *t*(195) = 4.77, *p* < 0.001] and indulgent homes [girls: *M* = 3.70, *SD* = 0.50; boys: *M* = 3.48, *SD* = 0.50, *t*(171) = 2.61, *p* < 0.01].

### Mediating Effects

**FIGURE 1:**
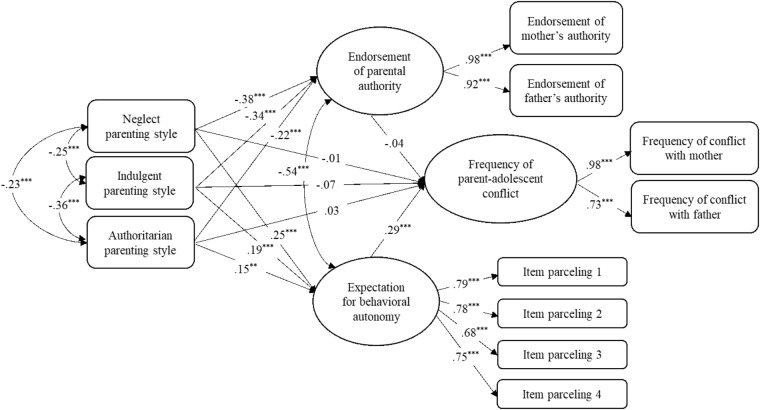
Adolescents’ expectation for autonomy and beliefs about parental authority as mediators between parenting styles and the frequency of parent-adolescent conflict. Standardized path coefficients are presented in the model. ^∗^*p* < 0.05; ^∗∗^*p* < 0.01; ^∗∗∗^*p* < 0.001.

**FIGURE 2:**
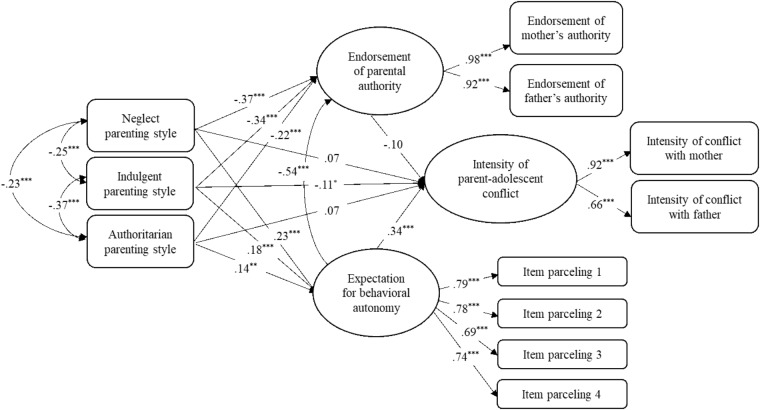
Adolescents’ expectation for autonomy and beliefs about parental authority as mediators between parenting styles and the intensity of parent–adolescent conflict. Standardized path coefficients are presented in the model. ^∗^*p* < 0.05; ^∗∗^*p* < 0.01; ^∗∗∗^*p* < 0.001.

**FIGURE 3:**
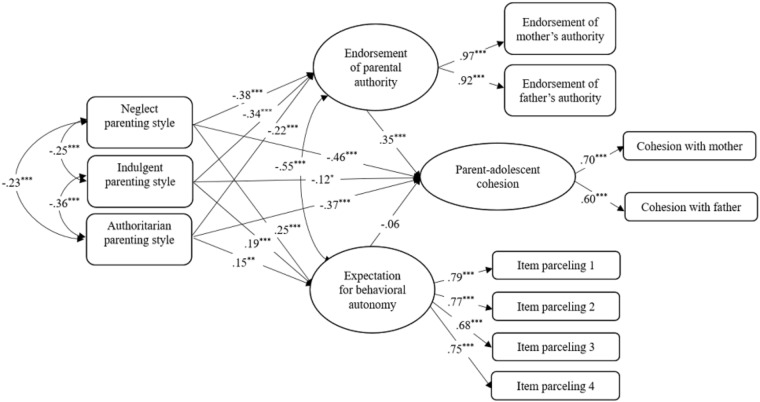
Adolescents’ expectation for autonomy and beliefs about parental authority as mediators between parenting styles and parent-adolescent cohesion. Standardized path coefficients are presented in the model. ^∗^*p* < 0.05; ^∗∗^*p* < 0.01; ^∗∗∗^*p* < 0.001.

To test our second hypothesis that expectations for behavioral autonomy and beliefs in the legitimacy of parental authority would mediate the links between parenting style and parent-adolescent conflict and cohesion, we used structural equation modeling in Mplus 7.4 (Figures 1–3, for the analyses of conflict frequency, conflict intensity, and cohesion, respectively). SES and grade were included as covariables. The categorical parenting style variable was represented as three dummy-coded variables with authoritative parenting as the reference category. Because the autonomy expectations scale had many items, we used a common parceling technique to estimate a highly reliable latent construct for that variable by randomly assigning items into four nearly equal-sized sets of indicators (Little et al., 2002). Finally, latent variables were constructed (using mother and father scales as indicators) for the conflict and cohesion variables, as well as the attitudes about legitimate parental authority variable. All models showed good fit with the data [conflict frequency: χ^2^ = 160.99, *df* = 56, CFI = 0.96, TLI = 0.95, RMSEA = 0.055; conflict intensity: χ^2^ = 167.23, *df* = 56, CFI = 0.96, TLI = 0.94, RMSEA = 0.058; cohesion: χ^2^ = 192.55, *df* = 56, CFI = 0.95, TLI = 0.93, RMSEA = 0.063).

In all three models, adolescents raised in neglectful, indulgent and authoritarian homes (compared to authoritative) reported lower level of beliefs about parental authority and higher expectations for behavior autonomy. Regarding frequency (Figure 1) and intensity (Figure 2) of conflict, greater expectation of autonomy was linked with more frequent and intense conflict, whereas regarding parent–adolescent cohesion (Figure 3), greater endorsement of authority was linked with greater relationship cohesion. Also, conflict intensity was lower for youth with indulgent parents and cohesion was lower for youth with neglectful, indulgent or authoritarian (compared to authoritative) parents.

Significance of indirect effects was computed using bootstrapping with 1000 resamples. A bias-corrected bootstrapped 95% confidence interval (CI) showed significant indirect effects from neglectful, indulgent and authoritarian parenting style to the frequency and the intensity of parent-adolescent conflict via adolescents’ expectation for behavior autonomy. For conflict frequency, 95% CIs were [0.033,0.126], [0.022,0.102], and [0.014,0.092] for neglectful, indulgent and authoritarian parents, respectively. For intensity of conflict, 95% CIs were [0.042,0.131] [0.027,0.105], and [0.019,0.097] for neglectful, indulgent and authoritarian parents, respectively. There also were significant indirect effects to cohesion via adolescents’ beliefs in the legitimacy of parental authority. The 95% CIs were [-0.202, -0.081], [-0.185, -0.071], and [-0.128, -0.0341] for neglectful, indulgent and authoritarian parents, respectively.

### Moderating Effect of Adolescents’ Gender

**FIGURE 4:**
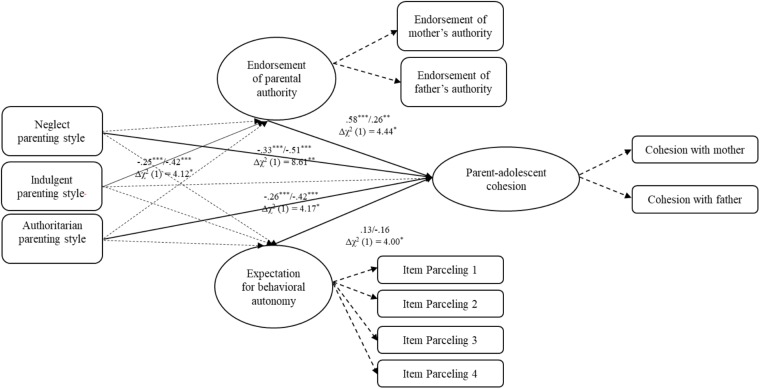
Results of multiple-group structural equation model evaluating the relationships of adolescents’ expectation for behavioral autonomy, their endorsement of parental authority and parent–adolescent cohesion across genders. Standardized path coefficients are presented in the model. Covariances, correlations and residuals are not shown. Solid lines indicate the pathway parameters are different between male sample and female sample. Dotted lines indicate the pathway parameters are similar between male sample and female sample. ^∗^*p* < 0.05; ^∗∗^*p* < 0.01; ^∗∗∗^*p* < 0.001.

Given possible gender differences in paths, we conducted multiple-group analyses. We had hypothesized that the links between parenting style and parent-adolescent conflict and cohesion would be stronger for girls than boys; we did not have hypotheses regarding the mediators however. Chi-square difference statistic (Δχ^2^) were used to compare fit between models. All structural paths were constrained to be equal for boys and girls and the overall model fit was compared to a model without any constraint. For conflict frequency and intensity, the unconstrained and fully constrained models were not significantly different—suggesting no gender moderation [Δχ^2^(11) = 14.88, Δχ^2^ (11) = 14.96, *p*s* >* 0.05]. In contrast, for cohesion, the unconstrained model provided a significantly better fit than the constrained model [Δχ^2^(11) = 23.45, *p* < 0.05]. To interpret this, we compared path coefficients for boys and girls one by one (see Figure 4). The negative prediction of cohesion from neglectful and authoritarian parenting (relative to authoritative parenting) was stronger for girls than boys; this was consistent with our hypothesis. As for the exploration of gender differences in the mediation paths, we found that the negative link between indulgent parenting style and parental authority was stronger for girls than boys, whereas the positive link between endorsement of parental authority and cohesion was stronger for boys than girls.

## Discussion

In the current study, we tested the associations between parenting styles and parent-adolescent relationships (Aim 1), examined the mediating effects of adolescents’ expectation for behavior autonomy and their endorsement of parental authority on these associations (Aim 2), and also explored the moderating effect of adolescents’ gender (Aim 3) in a sample of adolescents from mainland China.

### Parenting Styles and Relationships With Adolescents

In studies of Western families, parenting styles are recognized as having predictable associations with parent-adolescent conflict and cohesion. Previous studies have reported that adolescents of authoritative parents have lower conflict frequency and intensity and higher cohesion than those of authoritarian parents (Smetana, 1995; Assadi et al., 2011; Nelson et al., 2011; Sorkhabi and Middaugh, 2014). In contrast to previous research, the present study showed that adolescents reported similar levels of parent-adolescent conflict *frequency* regardless of parenting style. This result may be attributed to the traditional Chinese culture, which places emphasis on keeping harmonious relationships and avoiding confrontation (Peterson et al., 2005). This unique cultural context may alleviate any links between parenting and frequency of conflict because Chinese adolescents may avoid conflict with their parents.

However, conflict *intensity* did show associations with parenting style. Compared with indulgent parenting styles, adolescents of neglectful and authoritarian parents experienced greater intensity of conflict. Indulgent parents place relatively few demands on the adolescents’ behavior, giving them high degree of freedom to act as they wish. In contrast, neglectful parents are characterized as lacking warmth and guidance, whereas authoritarian parents place a high value on obedience and conformity and allow less verbal give-and-take. Conflict may be more intense in neglectful parenting style because the adolescent is making demands on a parent who otherwise is withdrawn and minimizing of the youth’s needs. Also, adolescents may be dissatisfied with authoritarian parents’ setting broad rules without emotional support, which leads to more intense conflict when it occurs. Other variables might also explain the effect. For instance, adolescents with neglectful parents are more likely to engage in delinquent behaviors (You and Lim, 2015), which itself may lead to more intense conflict.

In addition, the current study found that adolescents raised in authoritative and authoritarian parenting style reported similar levels of conflict intensity with parents. This is inconsistent with previous findings, which showed that Western adolescents raised in authoritarian parenting homes reported more intense parent–adolescent conflict than those raised in authoritative parenting homes (Smetana, 1995). One explanation for this difference in results may be that in Chinese culture, similar to training and tiger parenting, the motivation and intention of authoritarian parenting is to supervise children and promote optimal development, instead of simply controlling them (Chao, 1994; Kim et al., 2013). And Chinese adolescents may perceive positively the parents’ intention to supervise their development, resulting in no direct association between levels of parental control and conflict intensity.

With regard to parent–adolescent relationship cohesion, the current study showed that adolescents with authoritative parents reported the highest levels of cohesion. This result extends previously published work in various cultural groups showing greater cohesion for authoritative parenting (e.g., Nelson et al., 2011). Authoritative parenting is characterized by a high degree of warmth and acceptance as well as supervision, but also including the granting of adolescent autonomy (Baumrind, 2005). In Chinese and Western cultures today, adolescents seek greater independence along with support (compared to children)—a balance of youth and parent goals that is best met in authoritative households that promote close relationships. In contrast, neglectful parents’ lack of warmth and supervision, which may be interpreted as irresponsibility, may hinder the establishment of cohesive relationships. Indulgent and authoritarian parents provided either limited guidelines or limited support for their children. All these characteristics were likely to reduce parent–adolescent cohesion.

### Expectation for Behavioral Autonomy

Our second aim was, in part, to identify potential mediating effects of adolescents’ expectations for autonomy. Results showed that adolescents’ autonomy expectations mediated the links between parenting styles and both the frequency and intensity of parent–adolescent conflict. Specifically, compared to adolescents in authoritative homes, those in neglect, indulgent, and authoritarian homes reported stronger expectations for autonomy, which in turn were linked with more frequent and intense parent-adolescent conflict. This result was consistent with other studies which explored the relationships between parenting styles, adolescents’ expectation for behavioral autonomy and parent-adolescent conflict (Baumrind, 1991; Bush and Peterson, 2013).

Adolescents in authoritative families reported the lowest expectation for behavioral autonomy. This result may be due to that adolescents with authoritative parents have achieved appropriate autonomy, therefore, their desire to acquire more autonomy is not so strong. The salutary effect of authoritative parenting style on adolescents’ behavioral autonomy likely reflects the successful attainment of a socialization goal among authoritative parents: to facilitate autonomy and promote self-reliance. This socialization goal is accomplished by respecting their children’s needs and recognizing that adolescents legitimately have the right to control some aspects of their lives (Bush and Peterson, 2013).

Compared with authoritative parenting style, non-authoritative parenting styles have some characteristics that are thought to hinder the development of adolescents’ behavioral autonomy. Authoritarian parents are characterized as using hostile control or harsh punishment in an arbitrary manner to gain obedience and conformity (Bush and Peterson, 2013). At the same time, authoritarian parents provide limited warmth and responsiveness. In that context, adolescents are more likely to seek greater behavioral autonomy because it is not available to them. Also, indulgent and neglect parents provide few if any rules or discipline. Without sufficient firm control in the form of parental monitoring and guidance, adolescents raised in indulgent and neglect parenting families are more likely to experience high levels of independence before they can manage it themselves (Bush and Peterson, 2013). Also, adolescents in neglectful families lack parental supportiveness and those in indulgent homes are simply spoiled. Such adolescents may have high levels of autonomy, but it is not likely to have been developed through a healthy developmental process with their parents in a way that balances their growing self-determination and connectedness with their parents.

In agreement with previous research (Laursen and Collins, 2009), the current results revealed that adolescents’ expectation for behavioral autonomy statistically predicted greater parent–adolescent conflict—perhaps because parents favor less autonomy than do their teenage children. This parent-youth discrepancy has been found in individualistic and collectivist cultural groups within the United States and in other countries (Smetana, 1988; Pérez et al., 2016). Researchers have interpreted the discrepancy as a developmental phenomenon, in which adolescents’ need for autonomy exceeds parental concerns with maintaining order and protecting their children from harm (Jensen and Dost-Gözkan, 2015).

### Legitimacy of Parental Authority

The second mediating effect that was tested involved adolescents’ beliefs in the legitimacy of parental authority; results suggested some evidence for this effect. Compared with authoritative parenting, non-authoritative parenting was negatively associated with adolescents’ beliefs in the legitimacy of parental authority, which in turn were positively related to parent-adolescent cohesion. This finding is consistent with previous research (Fuligni, 1998; Darling et al., 2005; Assadi et al., 2011; Trinkner et al., 2012). Our interpretation is that with increasingly adult-like social cognitions and relationships, adolescents increasing question parental authority as they shift from unquestioning compliance to rational assessment with conditional obedience. Compared to other types of parents, authoritative parents, are more successful with continually renegotiating parental authority as their children “grow up,” because they use reasoning and explanations and are responsive to adolescents’ perspectives. This ongoing negotiation provides a context for parents and children to articulate and discuss divergent perspectives, which helps legitimize the parents’ authority by rationally justifying the boundaries of adolescents’ personal jurisdiction.

In contrast, authoritarian parents exert strict and sometimes arbitrary punishment without explanation. Also, they construct the boundaries of parental authority much more broadly than authoritative parents, which promotes resistance in adolescence (Smetana, 1995; Baumrind, 2005). In this context, adolescents struggle to internalize the legitimacy of parental authority. Also, in contrast to authoritative parents, indulgent and neglectful parents provide little information about boundaries or appropriate behavior. Such lax control can undermine parental authority, so that youth increasingly regard parents as not playing an authority role.

Parents who exercise their authority are satisfied when their adolescent children respect them, which helps maintain harmonious relationships in the family (Zhang et al., 2006; Jensen and Dost-Gözkan, 2015). As child-rearing agents, providers of information and rules, and primary sources of support for their children, parents need to establish their authority to better play their parenting roles. However, this occurs in a relationship context with adolescent, and the teenager’s endorsement of parents’ authority helps the adults meet their psychological needs as well. In such families, parents and youth consider each other’s boundaries and areas of control through negotiation and mutual respect, which builds more cohesive relationships.

In the current study, although adolescents’ expectations for behavioral autonomy and beliefs in the legitimacy of parental authority are both critical attitude domains, their mediating effects were different: autonomy expectations mediated the effect of parenting style on parent-adolescent conflict, but authority legitimacy mediated the effect of parenting style on parent-adolescent cohesion. Certainly, although they are correlated, conflict and cohesion delineate different aspects of parent–adolescent relationships (Zhang et al., 2006)—and, each may be affected differently by levels of parental authority and adolescent autonomy. The distinction may be particularly strong in Chinese culture which emphasizes conformity and obedience (Peterson et al., 2005). Parent-adolescent conflict was more likely to be linked with adolescents’ higher expectations for behavioral autonomy which runs against cultural norms, but cohesion was more likely to be linked with adolescents’ greater endorsement of parental authority which is consistent with cultural norms.

### Adolescent Gender

Our final aim was to test the hypothesis that the direct link between parenting style and relationship qualities would be stronger for girls than boys—and, to also explore whether there were gender differences in the mediating effects via adolescent autonomy and authority attitudes. The results indicated only a few such effects. Briefly, girls in authoritative and indulgent homes reported more cohesion with mothers than boys, and girls of neglect and authoritarian parenting reported lower level of parent–adolescent cohesion than boys. This may be due to that girls are more responsive and sensitive to social bonds than boys, and that cohesion and parenting style both reflects emotional atmosphere. Therefore, the relationships between parenting styles and cohesion were stronger for girls. Besides, girls of indulgent parents were less likely to endorse parental authority than boys, while endorsement of parental authority had greater effect on parent-adolescent cohesion for boys than girls. To the extent that parents normally set more rules and expect greater obedience of parental authority for girls than boys (Darling et al., 2005; Zhang and Fuligni, 2006), and consequently girls of indulgent parents may be more likely to feel that their parents did not shoulder the responsibility of cultivating them or establish the authority, given indulgent parents did not provide enough supervision and rules. Therefore, girls of indulgent parents endorsed lower level of parental authority. At the same time, since parents expected less conformity and obedience for boys, their endorsement of parental authority was more likely to live up to parents’ expectation, which may improve relationships with parents.

Although gender moderated a few paths in the direct and mediating models, overall, the majority of paths were not significantly different for boys and girls across all of the models that were tested. This may be due to that, with the implementation of the one child policy, Chinese parenting styles and socialization practices are becoming increasingly similar for their sole children (Lu and Chang, 2013), resulting in more similar associations between parenting styles and parent–adolescent relationships and also the mediating effects of autonomy and authority for these relationships for boys and girls.

### Limitations and Conclusions

Several limitations of this study should be noted. First, the participants were urban adolescents in mainland China which is characterized as collectivist culture, so generalizing the results to other cultures or groups should be done with caution. Second, the correlational design does not permit causal inferences. Longitudinal experimental data are necessary to identify causal relationships among the variables. Finally, we relied on adolescents’ self-reports. Previous research found that there were discrepancies between parents’ and youth’s perceptions on these variables (e.g., Jensen and Dost-Gözkan, 2015), so our findings may not represent what would be found using parents’ reports or observers’ ratings.

Despite these limitations, the current study has important implications. To our knowledge, this is the first study to examine the mediating effects of adolescents’ expectations for behavioral autonomy and beliefs in the legitimacy of parental authority, on the links between parenting styles and parent-adolescent conflict and cohesion. The findings of this study extend existing research and suggest that prevention and intervention efforts are needed to primarily target the reduction of non-authoritative parenting styles, and the promotion of acquiring appropriate levels of autonomy expectations and endorsement of parental authority. Future research should examine other possible mediating paths and sample a wider range of cultural contexts to explore adolescent development and family functioning.

## Ethics Statement

This study was carried out in accordance with the recommendations of the Institutional Review Board of Shandong Normal University. All subjects gave written informed consent in accordance with the Declaration of Helsinki. The protocol was approved by the Institutional Review Board of Shandong Normal University.

## Author Contributions

XB conducted the analysis and drafted the manuscript. YY and HL helped in performing the statistical analysis. MW coordinated the data collection and helped in the statistical analysis. WZ conceived and coordinated the study and helped to draft the manuscript. KD-D helped to draft the manuscript. All authors read and approved the final manuscript and the byline order of authors.

## Conflict of Interest Statement

The authors declare that the research was conducted in the absence of any commercial or financial relationships that could be construed as a potential conflict of interest.
