# Supplementary material for: Parenting Styles and Parent–Adolescent Relationships: The Mediating Roles of Behavioral Autonomy and Parental Authority
Source: Front Psychol. 2018 Nov 13;9:2187. doi: 10.3389/fpsyg.2018.02187 (PMC6243060; doi:10.3389/fpsyg.2018.02187)
Supplement: Supplementary file 1 [file Table_1.DOCX]

**Online Supplemental Material**

**Measurement Invariance Across Gender**

We specified a series of confirmatory factor analysis (CFA) models to examine measurement invariance across gender. We tested the equivalence of the scale properties (configural, loading, and intercept invariance) to demonstrate measurement invariance of the scales. Model fit indices and model comparisons can be seen in Table S1. To test the adequacy of model fit, we used the RMSEA, the comparative fit index (CFI) and the Tucker-Lewis index (TLI). A model is judged to be acceptable if the CFI and the TLI are both greater than .90 and the RMSEA is less than .08. Model invariance was assumed if (a) the overall model fit is acceptable, and the (b) the difference in model fit is negligible, specifically, the ΔCFI < .02 (Vandenberg & Lance, 2000).

For all measurements, the baseline model (M1) showed acceptable model fit indices, suggesting good construct validity of the measure. After constraining the factor loadings to be equal across gender (M2), no discernible differences were detected (ΔCFIs < .02), indicating that the loading invariance was established. All measurements also showed intercept invariance across gender. When constraining the item intercepts to be equal across gender (M3), no significant changes in model fit indices were found compared to M2 (ΔCFIs < .02).

Table S1

Model Fit Indices of the Nested CFA Models

| Models | χ^2^ | *df* | *p* | RMSEA | | TLI | CFI | ΔCFI |
| --- | --- | --- | --- | --- | --- | --- | --- | --- |
| Parenting styles |  |  |  | |  |  |  |  |
| M1: Baseline model | 364.84 | 176 | <.001 | | 0.045 | 0.900 | 0.917 |  |
| M2: Loading invariance | 388.124 | 189 | <.001 | | 0.058 | 0.905 | 0.915 | 0.002 |
| M3: Loading and intercept invariance | 446.732 | 202 | <.001 | | 0.062 | 0.891 | 0.895 | 0.020 |
| Conflict frequency with mother |  |  |  | |  |  |  |  |
| M1: Baseline model | 320.872 | 206 | <.001 | | 0.042 | 0.919 | 0.931 |  |
| M2: Loading invariance | 353.415 | 221 | <.001 | | 0.044 | 0.913 | 0.920 | 0.011 |
| M3: Loading and intercept invariance | 382.449 | 236 | <.001 | | 0.044 | 0.910 | 0.911 | 0.009 |
| Conflict frequency with father |  |  |  | |  |  |  |  |
| M1: Baseline model | 305.235 | 206 | <.001 | | 0.039 | 0.929 | 0.939 |  |
| M2: Loading invariance | 307.386 | 221 | <.001 | | 0.035 | 0.942 | 0.947 | -0.008 |
| M3: Loading and intercept invariance | 334.911 | 236 | <.001 | | 0.036 | 0.938 | 0.939 | 0.008 |
| Conflict intensity with mother |  |  |  | |  |  |  |  |
| M1: Baseline model | 326.444 | 193 | <.001 | | 0.047 | 0.904 | 0.922 |  |
| M2: Loading invariance | 357.008 | 208 | <.001 | | 0.048 | 0.900 | 0.913 | 0.009 |
| M3: Loading and intercept invariance | 383.225 | 222 | <.001 | | 0.048 | 0.899 | 0.906 | 0.007 |
| Conflict intensity with father |  |  |  | |  |  |  |  |
| M1: Baseline model | 380.059 | 193 | <.001 | | 0.055 | 0.881 | 0.905 |  |
| M2: Loading invariance | 396.397 | 208 | <.001 | | 0.054 | 0.889 | 0.904 | 0.001 |
| M3: Loading and intercept invariance | 431.327 | 222 | <.001 | | 0.055 | 0.885 | 0.893 | 0.011 |
| Cohesion with mother |  |  |  | |  |  |  |  |
| M1: Baseline model | 111.449 | 64 | <.001 | | 0.048 | 0.947 | 0.963 |  |
| M2: Loading invariance | 118.866 | 73 | <.001 | | 0.045 | 0.955 | 0.964 | -0.001 |
| M3: Loading and intercept invariance | 140.305 | 82 | <.001 | | 0.047 | 0.950 | 0.954 | 0.010 |
| Cohesion with father |  |  |  | |  |  |  |  |
| M1: Baseline model | 135.342 | 64 | <.001 | | 0.059 | 0.943 | 0.960 |  |
| M2: Loading invariance | 151.715 | 73 | <.001 | | 0.058 | 0.945 | 0.956 | 0.004 |
| M3: Loading and intercept invariance | 166.605 | 82 | <.001 | | 0.057 | 0.948 | 0.952 | 0.004 |
| Expectation for behavioral autonomy |  |  |  | |  |  |  |  |
| M1: Baseline model | 231.50 | 96 | <.001 | | 0.067 | 0.925 | 0.945 |  |
| M2: Loading invariance | 237.504 | 107 | <.001 | | 0.062 | 0.935 | 0.947 | -0.002 |
| M3: Loading and intercept invariance | 276.442 | 116 | <.001 | | 0.066 | 0.926 | 0.935 | 0.012 |
| Endorsement of parental authority |  |  |  | |  |  |  |  |
| M1: Baseline model | 1225.97 | 567 | <.001 | | 0.061 | 0.931 | 0.940 |  |
| M2: Loading invariance | 1245.803 | 591 | <.001 | | 0.059 | 0.935 | 0.940 | 0.000 |
| M3: Loading and intercept invariance | 1316.897 | 615 | <.001 | | 0.060 | 0.933 | 0.936 | 0.004 |
